# Adaptation by Men to the Nurse Role. "Being Craftsmen in the Construction of their Professional Trajectory"*

**DOI:** 10.17533/udea.iee.v40n3e12

**Published:** 2023-02-13

**Authors:** Sandra Milena Velásquez Vergara, María Elisa Moreno Fergusson, Edgar Orlando Arroyave Álvarez, Jasmín Viviana Cacante Caballero

**Affiliations:** 1 Nurse, PhD candidate. Professor. Universidad de Antioquia, Medellín (Colombia)E-mail: sandra.velasquez3@udea.edu.co Universidad de Antioquia Universidad de Antioquia Medellín Colombia sandra.velasquez3@udea.edu.co; 2 Nurse, PhD. Professor. Universidad de la Sabana, Chía (Colombia) Email: mariae.moreno@unisabana.edu.co Universidad de la Sabana Universidad de la Sabana Chía Colombia mariae.moreno@unisabana.edu.co; 3 Psychologist, PhD. Professor. Universidad de Antioquia, Medellín (Colombia)E-mail: edgar.arroyave@udea.edu.co Universidad de Antioquia Universidad de Antioquia Medellín Colombia edgar.arroyave@udea.edu.co; 4 Nurse, PhD. Professor. Universidad de Antioquia, Medellín (Colombia)E-mail: jasmin.cacante@udea.edu.co Universidad de Antioquia Universidad de Antioquia Medellín Colombia jasmin.cacante@udea.edu.co

**Keywords:** men, masculinity, gender identity, education, nursing, nurses, male, professional practice., hombres, masculinidad, identidad de género, educación en enfermería, enfermeros, práctica profesional., homens, masculinidade, identidade de gênero, educação em enfermagem, enfermeiros, prática profissional.

## Abstract

**Objective.:**

This work sought to describe the adaptation process by men to the nurse role.

**Methods.:**

Secondary analysis of data from a collective case study that had as participants 12 male nurses working in the city of Medellín, with ages between 28 and 47 years and average time of professional experience of 11 years. Information collection was carried out through in-depth interviews. The analysis was conducted through Roy’s Adaptation Model (RAM), reading of the interviews, identification of RAM’s components, grouping of fragments, assignment of tags, construction of a matrix and classification.

**Results.:**

The analysis performed accounts for the coping processes and adaptation by male nurses and the ineffective responses (control of emotions and emotional silencing) when practicing a role considered feminine.

**Conclusion.:**

In this study, it was possible to establish that, to achieve adaptation within nursing, men use strategies related with changes in bodily appearance, management of physical strength, and management of emotions.

## Resumen

**Objetivo.:**

Describir el proceso de adaptación de los hombres al rol enfermero.

**Métodos.:**

Análisis secundario de los datos de un estudio de casos colectivo que tuvo como participantes a 12 enfermeros que trabajan en la ciudad de Medellín, con edades entre los 28 y los 47 años y un tiempo de experiencia profesional promedio de 11 años. La recolección de la información se realizó mediante entrevistas en profundidad. El análisis se realizó a partir del Modelo de Adaptación de Roy (RAM), lectura de las entrevistas, identificación de los componentes del RAM, agrupación de fragmentos, asignación de etiquetas, construcción de una matriz y clasificación.

**Resultados.:**

El análisis realizado da cuenta de los procesos de afrontamiento y adaptación de los enfermeros y las respuestas inefectivas (control de emociones y silenciamiento emocional) al ejercer un rol considerado femenino.

**Conclusión.:**

En este estudio fue posible establecer que, para alcanzar una adaptación dentro de la enfermería, los hombres utilizan estrategias relacionadas con cambios en la apariencia corporal, el manejo de la fuerza física y el manejo de las emociones.

## Resumo

**Objetivo.:**

Descrever o processo de adaptação do homem à função de enfermagem.

**Métodos.:**

Análise secundária de dados de um estudo de caso coletivo que teve como participantes 12 enfermeiros que atuam na cidade de Medellín, com idade entre 28 e 47 anos e tempo de experiência profissional média 11 anos. As informações foram coletadas por meio de entrevistas em profundidade. A análise foi realizada por meio do Modelo de Adaptação de Roy (RAM), leitura das entrevistas, identificação dos componentes do RAM, agrupamento de fragmentos, atribuição de rótulos, construção de matriz e classificação.

**Resultados.:**

A análise realizada dá conta dos processos de enfrentamento e adaptação das enfermeiras e das respostas ineficazes (controle das emoções e silenciamento emocional) ao exercer um papel considerado feminino.

**Conclusão.:**

Neste estudo foi possível constatar que, para conseguir uma adaptação dentro da enfermagem, os homens utilizam estratégias relacionadas a mudanças na aparência corporal, manejo da força física e manejo emocional.

## Introduction

The concept of role has different definitions in disciplines, like psychology, sociology, anthropology, philosophy, among others; in this sense, the terminology used can vary. Considering the foregoing, it must be clarified that, this text, retook the sociological definition of role, which refers to the guidelines and behaviors society imposes and expects from an individual under certain situations; nevertheless, regarding the social expectations derived from the role each individual plays, these face a series of contradictions and ambiguities, characteristic of social systems and of their concrete operations.(1) Particularly, in the sexual division of work, said contradictions are related with stereotypes, given that while masculinity is associated with strong, extenuating, unhealthy and often dangerous activities, femininity is equivalent to light, delicate and clean work, which demands patience, abnegation, and dedication.(2) This conception has permeated, to a great extent, the bases of a gender system that has accompanied the health professions, specifically nursing, where the male presence has been proportionally lower.(3) 

For a male, performing a role considered historically feminine is not an easy task, given that it goes against the roles and functions socially accepted by the sex-gender system, which represents the binary and grants distinct qualities to men and women; an example of this is the traditional reason-emotion dichotomy to name that considered masculine or feminine. In this sense, it is expected for men to demonstrate publicly technical, physical, and rational competences, while empathy, compassion, and commitment related with caring for another human being, as in nursing, are actions that can be interpreted as signs of weakness, frailty, and lack of character.(4) In an heteronormative society, men who choose nursing as a professional option are exposed to not only a permanent justification of their decision, but also to shame and social signaling due to performing a role that supposedly does not correspond with what is established.(5) The aforementioned, according with Axel Honneth,(6) degrades the social value of certain forms of self-realization, with consequences in the self-esteem and self-confidence of those engaged in practices different from those assigned socially. Hence, the importance of knowing the different stimuli to which men are exposed from the moment they decide to study nursing and during the professional training with the objective of favoring work adaptation.

According with Callista Roy,(7) adaptation is defined as the *process and result through which people think and feel as individuals or in groups, using awareness and conscious choice to create human and environmental integration*. For Roy, human beings are holistic adaptive systems in continuous interaction with a changing environment, whose point of interaction are the stimuli the author classifies into: focal (the most immediate in the individual’s conscience and which trigger a coping response); contextual (which contribute to the focal stimulus, but are not the center of attention or energy); and residual (which are environmental factors, whose influence on the individual’s current condition is unknown).(7) Said stimuli, according to Roy,(7) upon coming into contact with the person, trigger responses to the coping processes that can be adaptative or inefficient, which are manifested into four modes of adaptation: physiological, self-concept, function of the role, and interdependence, but due to their relevance with the phenomenon of interest in this study, only the third of these was addressed. This article was conducted with the objective of knowing the adaptation process by men to the nurse role.

## Methods

This text resulted from the PhD thesis by the principal researcher, titled “*The Role of Men in Nursing, its Recognition and the underlying Emotions in the Exercise of their Professional Practice in the City of Medellín”.* The approach method was the secondary analysis of data from the methodological proposal by Ruggiano and Perry, a strategy that permits examining questions different from the primary study and interpreting the information retrospectively and - in this sense - answering questions different from those of the principal research.(8) That is why, in light of Roy's theory, the researchers inquired on the antecedents linked to the election of the professional role, the experiences during the professional formation, and the adaptation strategies*.*

Selection of participants. The study had the participation of 12 male nurses between 28 and 47 years of age, with educational levels ranging from undergraduate to PhD training and with performance in different job areas. The sampling process was initially selective and intentional and was conducted through phone calls to the first eight male nurses who were exercising their profession in the city of Medellín (Colombia), which, through knowledge by the principal author, were known to be key participants who complied with the eligibility criteria: male gender with two or more years of professional experience. Only one male nurse was excluded because he reported that he was working outside the country. After this, and through the snow-ball strategy, according to which the first participants referenced others interested in being part of the study, four additional male nurses were invited to participate to refine the initial emerging themes, until reaching theoretical saturation.(9)

Data generation process. The data generation technique used was the in-depth interview. The meetings were held virtually due to the confinement imposed by the Colombian government starting 15 March 2020 because of the COVID-19 health contingency. This required prior coordination with each of the participants to schedule the interview, which was carried out through phone calls and teleconference meetings using the Google Meets platform, seeking optimal audio and video conditions to have a broad, sustained, fluid and natural dialogue. Each meeting lasted approximately one and a half hour. The interviews were audio recorded, with prior authorization from each participant through the informed consent, and later were transcribed literally in the Microsoft Word^®^ text processor, looking to do so as soon as possible.

Analysis and interpretation process. The analysis process began by reading each of the interviews to identify the thematic declarations by the participants to, therein, extract the relations among the themes.(10) The work obtained 1,511 descriptions as a result of the analysis of the 12 interviews, which derived into six themes upon completing the analysis process. The foregoing was conducted through a detailed reading and construction of a conceptual map, visual tool that supported data classification and permitted discovering the interrelations existing among the emerging themes. Upon completing the analysis of the first two themes, the authors reviewed the Adaptation Model by Callista Roy(7) and read the data collected during the interviews and field diaries of the primary study and extracted those fragments related with the adaptation process by the male nurses when choosing their profession and during their university formation. The selection of the fragments chosen was discussed by the whole research team and, then, the narrative excerpts were classified according to how they corresponded to the central components of Roy’s model: stimuli, coping processes and behaviors or responses of situations related with their professional role. 

Rigor criteria. The reflexive process was present during the interviews, generation, and data analysis. In this sense, the findings were reviewed permanently by the research team, to comply with the rigor criteria during the analysis in terms of credibility, transferability, and verifiability.(11)

Ethical considerations. The study was approved by the Research Ethics Committee of the Faculty of Nursing at Universidad de Antioquia, who classified it as minimum-risk research according to that established in Resolution N° 008430 of 1993 in Colombia.(12)

## Results

The study had the participation of 12 male nurses working in the city of Medellín, ranging in age between 28 and 47 years and average time of 11 years of professional experience (Table 1).

**Table 1:** Characterization of study participants

Code	Age	Years of experience	Last degree obtained	Work area
Participant 1	33	10	M.Sc. in Mental Health	Teaching
Participant 2	32	6	Male nurse	Clinical Care
Participant 3	47	21	M.Sc. Administration	Administrative
Participant 4	36	14	Ph.D. in Nursing	Teaching
Participant 5	38	16	Ph.D. in Nursing	Teaching
Participant 6	31	2	M.Sc. in Nursing	Clinical Care
Participant 7	45	9	M.Sc. in Epidemiology	Clinical Care
Participant 8	35	14	Male nurse	Clinical Care
Participant 9	38	11	Male nurse	Home care
Participant 10	44	5	Male nurse	Clinical Care
Participant 11	28	4	Male nurse	Community care
Participant 12	43	20	MSc. in Nursing	Administrative

**Figure 1:**
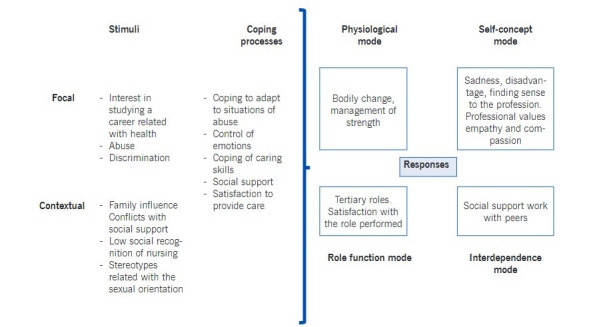
Adaptation process by the men to the role of male nurses

The adaptation process by the men to the role of male nurses was carried out in light of Roy’s Adaptation Model (RAM), which included stimuli, coping processes, and responses in the four adaptative modes (Figure 1).

The following describe the elements of Roy’s adaptation process.

### Stimuli

Upon analyzing the interviews, the focal stimuli identified three themes; the first of these was the **interest in studying a profession related with the health area**. This was expressed by one of the participants: I was always clear that the profession I was going to choose had to be one where I could have direct contact with people and be useful to society and in Nursing, I found that possibility (Participant 1). The other two stimuli identified were **abuse and discrimination**, which are related with the situations to which the participants were exposed in different social and academic scenarios for choosing a predominantly feminine career, which led them to even contemplating the idea of abandoning the profession. As expressed by one of the male nurses who during his academic formation had difficulties with one of his professors: That professor is very tough, very demanding, but quite primary to say things, and one day she swept the floor with me. That was something crazy, so much so that I almost quit […] (Participant 9). 

Similarly, another one of those interviewed faced disapproval from people close to him: when I said I had gone into nursing, the philosophy professor stated in public that he did not understand how a person with the opportunity to study something important had chosen to clean asses (Participant 11). In addition, the contextual stimuli identified four themes: family influence, conflicts with social support, social recognition of nursing, and social stereotypes. 

Family influence is related with the family influence on the professional choice, a situation considered by some with a genetic trait due to its hereditary nature. Illustrating the aforementioned, one of the study participants stated that nursing is something that is carried in the blood, like a family inheritance; it's like a legacy. In my family, we have people who have been dedicated to this [… ] (Participant 2). 

Another example of contextual stimuli is **conflicts with social support**, which is reflected on relating with friends, professors, and parents, who are unaware of the scope of nursing and have pejorative expressions in relation to it. One of the participants shared what he felt when he told one of his friends of his professional choice professional: *I had an ugly brush, the man is an engineer and he told me: why are you going to study that?, knowing that you as a male nurse will dedicate yourself to cleaning* [… ] *you can imagine what* (Participant 10). 

Likewise, another male nurse remembered that during the professional formation, he found resistance in female professors; *they told me openly: men are not good for that, they often stated* (Participant 9). In turn, another man interviewed said that, although his mother supported him during his academic formation, she did not agree with his professional choice, given her expectations focused on another trade: *I left the community, and I continued studying nursing and my mother did not like the idea because she wanted me to be religious* (Participant 7). Also, during the dialogues analyzed, it was established that another contextual stimulus is related with **low social recognition of nursing**. For one of the participants, *the nurse role in our society is regrettable, it is sad* […], *we had to be in the middle of a pandemic so that as professionals we were visible and valued a little more* […] (Participant 9). 

Lastly, in the contextual stimuli, the second reading of the interviews permitted identifying that for the participants **the stereotypes related to the sexual orientation** continue to be valid for choosing a profession like nursing: *at social level, when people see you as a male nurse, they generally judge you and ask themselves if your sexual condition is or isn’t defined,* get me*?, people ask if you are or are not, right away, they associate you with a homosexual individual* (Participant 8). A similar situation to that expressed by another male nurse: *a relation starts to be made of the discipline with certain stereotypes*, so [… ] *one hears out there, people saying: nursing is no longer only for women, nursing is also for gays, not to say the other word!* (Participant 5). 

### Coping processes

This section presents some situations faced by male nurses during different stages of their lives where nursing has been present and in which it may be seen how the coping process takes place in function of the adaptation to situations of abuse, control of emotions, development of care skills, search for social support and satisfaction by providing care.

With regard to **coping to adapt to situations of abuse**, one interviewee reported that since an early age he had to confront violence and poverty, which is why when choosing his profession, he thought: *I want to be a male nurse so I won’t be afraid to look at reality in the face* (Participant 11). In the same sense, another participant stated that one of the strategies to face abuse during his university formation was *to try to understand the women professors and not judge them, but he did see female professors who lived with a certain anger against men, like with resentment* (Participant 9). 

With respect to the **control of emotions** by the participants, one of the male nurses manifested that when he was studying, he was providing care to *an adult who was almost 90 years old with Alzheimer’s, I did not know what to do with that old man, and I started to cry* […]*; but the hardest part for me was that I felt I had to cry in silence*” (Participant 4). 

Related to the theme of **coping of care skills**, one of the participants expressed that during his training he developed skills for neonatal care defying his own fears; *I was very rough, and taking these children so young gave me a lot of difficulty, but I said: I have to learn to handle these little kids, and I learned to manage my strength and to manipulate them; for me, it was something quite significant because - in spite of my male condition - I had been able to do things that were like more delicate* (Participant 4). 

For **social support**, it was possible establish that during the professional formation, for men to adapt, they recur to social support and to the construction of empathic relations with classmates and professors. *Fortunately, I found professors who told me: you are capable, you have the same capacities. Go ahead!* (Participant 9). Along with the foregoing, it was possible to identify within the analysis **the satisfaction by providing care**. This was so expressed by one interviewee: *amid so much work, the most comforting thing happens when someone you don't know turns to you and says some nice words* [closes his eyes and sighs]*: you are like a son! What you experience and what you feel at those moments is a very beautiful thing* (Participant 10).

### Adaptative responses

During the analysis, it was found that men in nursing choose certain areas as adaptative response to the profession; this fact evidences not only adaptation to a role different from that allowed by hegemonic masculinity, but the persistence of the sexual division of labor within the profession. For two of those interviewed, *men look for areas where they can have certain power* (Participant 12), *they look for areas where their work is recognized more* (Participant 2). 

The **physiological mode** discovered that, to adapt to a specific role, some of the participants opted for changing their bodily structure, to have the musculature and sufficient strength to attend to situations of care that required it. One of them shared his experience: *about the physical strength, it is a generalized matter and I think that precisely due to that pressure I started to get fat, I was very skinny, and I would ask myself: how will I get on top of that patient to restrain him? So, I started to eat more at night* (Participant 1). Similarly, another participant reported that when he was a student, his role was related with physical strength: *for example, with an aggressive patient, bring the men to help restrain him! Or, if he is going to fight, to grab him; or, if the patient has to be moved: bring the men* [sarcastic tone of voice] [… ] (Participant 4). 

In the **self-concept mode**, the narratives by the participants evidence situations that caused feelings of sadness, worthlessness, and frustration, as well as a motive to reflect upon their lives and future plans focused on serving others: *I was in a hospital alone, with pain, cold,* […] *I spent the time crying and I would say: I don’t want other people to go through this situation, I want to be that person that can help others* (Participant 6).

In the **role function mode,** male nurses from the interaction with other professionals during their academic formation stage find sense to their professional choice and are seen as role models, independent of their gender. This happened to one of the male nurses: *my female professor put on gloves, stabilized the patient and got everyone working. When I saw the patient well, recovered, I looked at my professor and said: hey, that is what someday I imagined I wanted to see, that is what I want to be* (Participant 4). In that same sense, another participant recognized values and attitudes characteristic of the role of a male nurse. For him, *what really defines nursing professionals is the responsibility, honesty, and passion they have to place at the service of other people their knowledge and skills* (Participant 8). Together with the aforementioned, it was possible to identify within the analysis that the participants assume tertiary roles in the day-to-day, often associated with personal and social expectations around their roles as male nurses. (13) This was, thus, stated by one of the men interviewed: *in your work you have to act as a counselor, as a priest* [*smiles*]*, even as a doctor.* [… ] *to be able to help the patient* (Participant 10).

Among the adaptative responses identified during the analysis, there was evidence of the satisfaction experienced daily by male nurses, when their work is recognized: *It fills me with joy and satisfaction that they tell me: I love that nurse because he helped my grandma!* [… ] *It makes me vibrate by the fact of curing a patient, of helping another* (Participant 9). Similarly, one of his colleagues stated that for him the most important thing is *to provide quality care, for it to be a good experience for the person* (Participant 6). Another example of adaptative response to the role function was exemplified by one of the participants, who in his work trajectory has managed to perform in unconventional contexts for a male nurse, like working with homeless people: *as a professional, you have to be resourceful, my work was hard, it was necessary to be out in the sun, we did not have many elements to provide care: I would sometimes sit on a rock to care for the people* (Participant 11). In this report, fundamental attributes of the person are noted to meet the needs of users, such as courage to face difficulties, resourcefulness, and persistence. 

Finally, in the **interdependence mode**, the participants made known the support networks they had available when they chose nursing. For some of them, it was very significant to have the support from women close to them (mothers, sisters, female friends). One of the male nurses expressed it thus: *in my house, we are four siblings, my older sister is a respiratory therapist; she liked the idea and agreed that I should enter the university* (Participant 4). Another male nurse, is thankful for the support received from his mother; *she is the one who urged me most. I am what I am because of my mom* ; *that is the truth in my life* (Participant 10). To the aforementioned, we can add the statement by another interviewee: *looking precisely for networks and support, I have gone to the university because I still have female professors who are friends, because one only sees the possibility of accompaniment* (Participant11). 

With respect to interpersonal relations, some of the participants said that it is more difficult to work with female colleagues because they are emotional, complicate processes, and are competitive, as stated by this participant: *it is much easier to work with men than with women; male nurses are less emotional and less competitive than women* (Participant 2). In the same sense, another interviewee indicated that *a man does not complicate his life too much; that is even noticed during the change of shift* (Participant 6).

## Discussion

The second analysis performed in light of Roy’s theory permitted identifying within the focal stimuli that men - due to their professional election - continue facing social prejudice and stereotypes, which, according with Gugel and Lima,(14) are a product of discursive practices that tend to associate women as natural caregivers. These biases, according with Burguette M *et al*.,(15) not only bring as consequence exclusion and limited participation by men in nursing, but also contribute to perpetuate gender inequality.

Upon analyzing the participants’ experiences, according with that proposed by Roy,(7) it is possible to affirm that the situations of abuse during the training can generate ineffective responses, feelings of sadness and impotence that affect self-esteem, self-consistency, and self-concept, given that the last is formed from the combination of beliefs the subjects have of themselves and of the perceptions of others, thus, guiding individual behaviors. 

With respect to the abuse received by the students, Ayala(16) proposes that these could be explained, largely due to a condition called submissive aggression syndrome, where nurses guide actions of violence toward their disciples with the desire to achieve recognition and maintain a high professional standard within a competitive and voracious market.

Moreover, among the contextual stimuli, this study identified conflicts with social support, given that according to some of those interviewed, people close to them (parents, friends, professors) did not agree on their opting for a feminine profession. In relation with the foregoing, Peña *et al*.,(17) consider that barriers and social supports an individual receives, besides helping or hindering the possibility of reaching academic achievements and personal goals, influence upon the beliefs they have on their capacity to help successfully in a vocational field and in their aspirations to comply with the desired objective. 

Furthermore, Labra *et al*.,(18) indicate that men tend to specialize in areas of greater prestige and autonomy consistent with masculinity and avoid performing in areas associated with maternity and femininity, and instead, look for areas where they have little physical contact with others and access to technology with the purpose of masking feelings of compassion and dedication, which could be interpreted as a sign of weakness.(19) The findings permit affirming that although certain characteristics were evident that are related with hegemonic masculinity, the participants develop different coping strategies that allowed their promoting adaptation to their role of male nurses, which, according with Roy,(7) emerge from spontaneous responses that enable wellbeing, satisfaction, and adaptation in their contexts, which for the specific case of this study, was evidenced in the control of emotions, development of care skills, and search for social support.

Upon analyzing the responses in the adaptation modes proposed by Roy,(7) it was possible to determine in the participants, in the role function mode, changes related with bodily appearance, management of physical strength, and management of emotions. The foregoing, according with Ritxar Bacete,(20) is related with social beliefs from heteronormativity, which indicate that a true man has physical toughness and emotional control that translates into the prohibition of public manifestations of affection and into emotional silencing. Along with this, from the reports by those interviewed, it was possible to establish, in the role function mode - as proposed by Roy,(7) that to comply adequately with their role as male nurses, the participants choose tertiary roles, like acting as a priest, counselor, or physician, which are not only associated with social expectations, but also with the compliance of their responsibilities to provide comprehensive care.

Another important finding in the study regarding the self-concept mode is related with what nursing has inherited, which, according with historian Ana Luisa Velandia,(21) have influenced on its evolution, being precisely the female issue the most significant and which - in turn - has been linked to the values of the Catholic relationship, hence, female characteristics are strongly related with those expected of a nun: submission, obedience, abnegation, kindness, softness, subtlety, among others. 

Likewise, and with respect to self-concept, the participants - in spite of being from the male gender - have values, like empathy, compassion, and the desire to help others, which was evident even before entering the university. In this sense, supported on Sara Ahmed,(22) it may be stated that emotions are constructed in interaction with others and that these are not related with gender.

## Conclusion

After the secondary analysis in which RAM was used, it was possible to establish that for men to achieve adaptation within nursing, they reconcile their emotions in different spheres (family, academic, work), given that the fact of expressing them openly may be interpreted as sign of weakness or frailty. Likewise, the strategy used permitted identifying focal and contextual stimuli that can be interpreted as obstacles within the adaptation process to the role, not only during the professional formation stage, but also the work stage, due to the stereotypes that remain in effect at social level. In consideration with the aforementioned, it is pertinent to continue investigating about the difficulties of male nurses since their university formation to the exercise of their professional work and on the adaptation strategies they undertake during said stages, so that greater scientific evidence may be obtained to permit developing intervention strategies based on the experiences, needs, and expectations of the male nurses.

Study limitations. The COVID-19 pandemic limited the data collection to the virtual scenario, which did not permit detecting other contents, like non-verbal language in its natural environment. Moreover, considering the scope of the qualitative research and of the hermeneutic approach, the results are not generalizable in population terms, so we are invited to read the findings within the context and time in which they were constructed.
